# Necrology

**Published:** 1902-03

**Authors:** 


### NECROLOGY.

LOXLA EDWARDS, D.V.S., Assistant District Veterinarian, of Washington, D. C., died on February 25th, after an illness of four weeks of pneumonia. Dr. Edwards was a graduate of the United States College of Veterinary Surgeons, 1901.

The following resolutions were adopted by the Washington Veterinary Association, of which he was a fellow:

The following resolutions were adopted February 27, 1902, by the members of the Washington Veterinary Association in memory of their late friend and fellow, Dr. Loxla Edwards, who departed this life the morning of February 25th.

WHEREAS, The Good God has deemed it wise and expedient to take from our midst our esteemed and honored colleague, Dr. Edwards, late Assistant and House Surgeon of the United States College of Veterinary Surgeons, Washington, D. C.

*Resolved*, That by the death of Dr. Loxla Edwards we lose a most earnest and devoted member of the profession. Suddenly, when life seemed brightest and health vigorous and permanent, in the twinkling of an eye his soul passed into eternity, and his life, so filled with hopes of usefulness, sank to struggle no more with earth's waves and billows. His life seemed a truly noble one, and the loss is irreparable.

*Resolved*, That to the family of our deceased member we extend our heartfelt sympathy, earnestly praying that in this sad bereavement they may find consolation in the belief that he has joined his Creator within the portals of heaven.

*Resolved*, That a copy of these resolutions be spread on the minutes of this association and a copy be forwarded to the bereaved family.

MORRIS H. WALMER,

DR. J. C. HEIDE,

ADAM FISHER,

Committee.

ARTHUR AMES, V.S., of Saltsburg, Pa., a registered non-graduate of Washington County, died in December.

LINDSAY JONES, V.S., a non-graduate registered veterinarian, living at Simpson's Store, Washington County, Pa., died in the latter part of 1901.

---

COMMENTS ON THE DEATH OF DR. HUIDEKOPER.

I was sorry to see by the JOURNAL the death of your coworker. The kind things said by acquaintances indicate the esteem in which he was held. My sympathies are with you and all intimately connected with him.

J. S. GRANT, V.S., Portland, Mich.

I have just learned of the death of our colleague, Dr. R. S. Huidekoper, a true loss to the profession. It is not a long time since I had the pleasure of reading his lectures in part, enough though to appreciate his high qualities as a veterinarian. Accept my condolence.

A. I. TELMOSSE, V.S., St. Andre, Quebec.

It was with sincere regret and sorrow that I learned of the untimely death of my friend Dr. R. S. Huidekoper; he was a loyal friend and helped the advancement of the veterinary profession. He always had my respect, esteem, and help in his ardent endeavor to help the army veterinarian and the upbuilding of veterinarians. I am of the opinion that his illness was due to his noble work at Washington, for I know that a year ago, when the Army bill was before the House, he was ill then with pneumonia, and ran the greatest risk in his work at that time.

R. H. HARRISON, D.V.S., Milwaukee, Wis.

